# Half-dose glucarpidase as efficient rescue for toxic methotrexate levels in patients with acute kidney injury

**DOI:** 10.1007/s00280-021-04361-8

**Published:** 2021-10-20

**Authors:** Sandra Heuschkel, Theresa Kretschmann, Raphael Teipel, Simone von Bonin, Stephan Richter, Susanne Quick, Nael Alakel, Christoph Röllig, Ekaterina Balaian, Frank Kroschinsky, Holger Knoth, Martin Bornhäuser, Malte von Bonin

**Affiliations:** 1 Klinik-Apotheke, Universitätsklinikum Carl Gustav Carus, Technische Universität Dresden (TUD), Fetscherstrasse 74, 01307 Dresden, Germany; 2 Medizinische Klinik und Poliklinik 1, Universitätsklinikum Carl Gustav Carus, Technische Universität Dresden (TUD), Fetscherstrasse 74, 01307 Dresden, Germany; 3 German Cancer Consortium (DKTK), Partner Site Dresde, Dresden, Germany; 4 German Cancer Research Center (DKFZ), Heidelberg, Germany; 5 Medizinische Klinik und Poliklinik 3, Universitätsklinikum Carl Gustav Carus, Technische Universität Dresden (TUD), Fetscherstrasse 74, 01307 Dresden, Germany

**Keywords:** Half-dose glucarpidase, High-dose methotrexate, Acute kidney injury, Methotrexate plasma concentration, Folinic acid

## Abstract

**Purpose:**

High-dose methotrexate (HDMTX)-associated acute kidney injury with delayed MTX clearance has been linked to an excess in MTX-induced toxicities. Glucarpidase is a recombinant enzyme that rapidly hydrolyzes MTX into non-toxic metabolites. The recommended dose of glucarpidase is 50 U/kg, which has never been formally established in a dose finding study in humans. Few case reports, mostly in children, suggest that lower doses of glucarpidase might be equally effective in lowering MTX levels.

**Methods:**

Seven patients with toxic MTX plasma concentrations following HDMTX therapy were treated with half-dose glucarpidase (mean 25 U/kg, range 17–32 U/kg). MTX levels were measured immunologically as well as by liquid chromatography–mass spectrometry (LC–MS). Toxicities were assessed according to National Cancer Institute—Common Terminology Criteria for Adverse Events (CTCAE) v5.0.

**Results:**

All patients experienced HDMTX-associated kidney injury (median increase in creatinine levels within 48 h after HDMTX initiation compared to baseline of 251%, range 80–455%) and showed toxic MTX plasma concentrations (range 3.1–182.4 µmol/L) before glucarpidase injection. The drug was administered 42–70 h after HDMTX initiation. Within one day after glucarpidase injection, MTX plasma concentrations decreased by ≥ 97.7% translating into levels of 0.02–2.03 µmol/L. MTX rebound was detected in plasma 42–73 h after glucarpidase initiation, but concentrations remained consistent at < 10 µmol/L.

**Conclusion:**

Half-dose glucarpidase seems to be effective in lowering MTX levels to concentrations manageable with continued intensified folinic acid rescue.

**Supplementary Information:**

The online version contains supplementary material available at 10.1007/s00280-021-04361-8.

## Introduction

Methotrexate (4-amino-10-methylfolic acid, MTX, ametho-pterin) is structurally related to folic acid and its antineoplastic effect is considered to mainly rely on the competitive inhibition of dihydrofolate reductase (DHFR).

MTX is applied to treat hematological malignancies as well as solid tumors. Protocols containing high-dose (HD) MTX (typically defined as a single dose of ≥ 500 mg/m^2^ body surface area) are used in acute lymphoblastic leukemia (ALL), osteosarcoma, and lymphomas.

The vast majority of MTX is excreted via the kidneys. Free plasma MTX undergoes glomerular filtration and is actively secreted in the proximal tubules [1, 2]. In urine, MTX and its metabolites show better solubility under alkaline conditions, whereas acidity (pH < 7.0) promotes intratubular crystal formation [3, 4].

Incidence and severity of MTX toxicity correlate with exposure and are dependent on the concentration as a function of time (area under the curve). Changes in pharmacokinetics with delayed elimination have been linked to excess MTX toxicity [5]. Apart from folinic acid administration (5-formyltetrahydrofolic acid, FA) to rescue normal body cells from MTX toxicity, unspecific measures to maintain renal function like hyperhydration and avoidance of potentially nephrotoxic comedication are combined with more specific interventions to perpetuate MTX pharmacokinetics, e.g., urine alkalization, prevention of drug interactions, and drainage of third space fluids. Acute kidney injury (AKI) caused by HDMTX arises through crystal nephropathy, which occurs when MTX precipitates within the renal tubules [3, 6]. Despite appropriate supportive care, AKI develops in 2–12% of patients treated with HDMTX [4]. AKI is associated with reduced MTX elimination and prolonged exposure, which may lead to an excess in MTX-related morbidity and mortality [7]. Besides intensified FA rescue and hyperhydration, two treatment approaches to lower toxic MTX plasma concentrations are promoted in this situation: glucarpidase (carboxypeptidase G_2_, CPDG_2_, Voraxaze™) or high-flux hemodialysis, the latter being more invasive and less effective [4, 8]. Glucarpidase is a recombinant enzyme which rapidly hydrolyzes extracellular MTX into inactive metabolites, 4-deoxy-4-amino-N10-methylpteroic acid (DAMPA) and glutamic acid. Advocated in international guidelines and in most clinical protocols dealing with HDMTX, glucarpidase still has no European market approval. The recommended dose of glucarpidase is 50 U/kg; however this has not been formally established by a dose finding study in humans. Small numbers of case series suggest that glucarpidase might be effective at lower quantities [9–14]. Considering the high costs of the drug, dose reduction strategies also have the potential to significantly reduce the financial burden for this rescue treatment.

Here, we report our experience with half-dose glucarpidase in patients with HDMTX-associated AKI.

## Materials and methods

Since 2017, all patients treated with HDMTX who were at high risk for an excess MTX-associated toxicity were deemed eligible for half-dose glucarpidase rescue. Briefly, following *short-term* (≤ 4 h) infusions, MTX levels > 10, 1, and 0.1 µmol/L at 24, 48, and 72 h after the start of MTX infusion, respectively, and an increase in creatinine (> 25% above baseline within 24 h, > 50% within 96 h) were considered as potentially harmful. Thresholds in *prolonged infusions* (≥ 24 h) were MTX levels > 150, 3, 2, 1, and 0.25 µmol/L at 24, 36, 42, 48, and 54 h after HDMTX initiation, respectively, and an increase in creatinine (> 25% above baseline within 48 h). Monitoring and FA rescue protocols are detailed in the supplement. In osteosarcoma the FA rescue followed the normogram provided within the clinical trial protocol [15].

Warning signs for protracted MTX clearance and strong indicators for glucarpidase treatment in *short-term* and *prolonged* MTX infusions were applied according to Supplemental Tables 1 and 2. To emphasize, the decision for glucarpidase treatment was not necessarily based on a single laboratory result but on the dynamics of MTX clearance and renal function. Definite criteria for glucarpidase treatment were adapted from international recommendations [8].

All patients received adequate hydration (at least 3 L of intravenous fluids per day starting at least 12 h before MTX infusion), urine alkalization (target pH ≥ 7.0), and FA rescue (dosage dependent on MTX levels). Third spacing (volume ≥ 500 mL) like pleural effusion was removed before starting HDMTX. Drugs which might delay MTX elimination (e.g., proton pump inhibitors, penicillins, and cotrimoxazole) were stopped in advance. MTX levels were measured immunologically in-house (ARK™ Methotrexate Roche c-pack, Teva Pharmaceuticals USA Inc.) and used for FA dose calculation. Starting with a baseline sample prior to the administration of glucarpidase, MTX concentrations were also analyzed by liquid chromatography–mass spectrometry (LC–MS) in an external, certified laboratory. LC–MS results were only available with several days delay; therefore, could not be used for immediate clinical decision making.

A single dose of glucarpidase was administered to patients with toxic MTX levels and worsening renal function [7]. The drug was given as intravenous injection over 5 min at 25 Units per actual body weight (U/kg). The calculated dose was rounded to whole vial numbers. FA rescue was interrupted within 2 h before and after glucarpidase administration and continued until MTX clearance.

The impact of glucarpidase on MTX serum levels was regarded as primary objective. Therefore, the time axis in all graphs was normalized to glucarpidase administration. Rapid and sustained clinically important reduction (RSCIR) was defined as ≤ 1 µmol/L at 15 min after the initial injection lasting for at least 8 days [16].

The assessment of toxicities (nephro-, hepato-, hemato- and neurotoxicity) was the secondary objective and graded according to National Cancer Institute—Common Terminology Criteria for Adverse Events (CTCAE) v5.0.

The study has been approved by the Institutional Review Board and was conducted in accordance with the declaration of Helsinki (BO-EK-146032021).

## Results

### Patient characteristics

In the observation period a total of 173 patients received 571 doses of HDMTX of whom seven patients were treated with half-dose glucarpidase. Patient characteristics are summarized in Table 1.

**Table 1:** Patient characteristics, treatment and outcome measures

Patient number		#1	#2	#3	#4	#5	#6	#7
Baseline characteristics								
Age [years]		49	70	47	66	59	19	71
Gender [f/m]		f	m	m	m	m	m	m
BMI [kg/m^2^]		25.4	28.0	25.5	26.6	32.4	29.7	24.4
Diagnosis		PBL	DLBCL	BCP-ALL	PCNSL	r/r PCNSL	OS	PCNSL
Regimen		MARix [17]	MTX [18]	Cons. IV^a^	MATRix^b,c^	MATRix^c^ [19]	MTX [15]	MTX [17]
No. of MTX cycle		1	2	4	3	3	11	2
MTX dose and administration	[g/m^2^]	1.4^d^	1.5	1.5	3.5	3.5	12.0	3.5
	[g]	3.00	3.36	3.26	6.72	8.26	25.20	7.21
	[h]	3.25	3.25	24.00	3.25	3.25	4.00	3.25
Creatinine at baseline [µmol/L]		90	77	69	76	66	57	62
eGFR [mL/min/1.73 m^2^]		65	87	> 90	90	> 90	> 90	> 90
Dynamics of MTX and creatinine levels before CPDG_2_ administration								
MTX at 24–28 h [µmol/L]^e^		53	28	55	25	134	515	106
Creatinine at 16–24 h [µmol/L]		122	266	106	139	166	229	262
MTX at 48 h [µmol/L]^e^		34	n.a.	7	14	33	n.a.	41
Creatinine at 40–48 h [µmol/L]		162	398	216	267	226	244	344
CPDG_2_ administration and its immediate effects on MTX levels								
MTX level *before* CPDG_2_	[h to CPDG_2_]	14	5	4.3	9.7	0.3	0.5	0
	[µmol/L]^e^	34.0	10.6	3.1	14.0	19.0	182.4	30.4
CPDG_2_	[h after start of MTX]	62	47	70	58	60	42	54
	[U/kg]	24	31	22	25	17	32	24
	[U]	2000	3000	2000	2000	2000	3000	2000
MTX level *after* CPDG_2_	[h after CPDG_2_]	2.5	25.5	1.7	7.3	0.9	1.0	0.5
	[h after start of MTX]	64.5	72.5	72.0	n.d.	60.5	60.0	55.0
	[µmol/L]^f^	0.35	0.05	0.02	n.d.^g^	0.20	2.03	0.71
Maximum renal toxicity and neurotoxicity								
Creatinine [µmol/L]		219	702	235	322	226	344	344
Dialysis [y/n]		n (declined)	n	n	n	n	y	n
Neurotoxicity [CTCAE v5.0]		Unchanged	0	0	0	Unchanged	0	3

^#^Patient number, *BCP-ALL* B-cell precursor acute lymphoblastic leukemia*, Cons* consolidation, *CPDG*_*2*_ glucarpidase*, CTCAE v5.0* National Cancer Institute - Common Terminology Criteria for Adverse Events version 5.0*, DLBCL* diffuse large B-cell lymphoma, *eGFR* estimated Glomerular Filtration Rate based on Chronic Kidney Disease Epidemiology Collaboration (CKD EPI), *f* female, *m* male, *MTX* methotrexate, *n* no, *n.a.* not applicable (patient had already received glucarpidase), *n.d.* not determined, *OS* osteosarcoma, *PBL* primary breast lymphoma, *PCNSL* primary CNS lymphoma, *y* yes

^a^GMALL 08/2013 trial (EudraCT 2013- 003466- 13)

^b^IELSG43 trial (EudraCT 2012- 000620- 17)

^c^Discontinuation when MTX toxicity became evident

^d^Target dose 3.5 g/m^2^ which was reduced to 40% due to renal impairment

^e^Determined by immunoassay

^f^Determined by LC–MS

^g^5.9 µmol/L as determined by immunoassay

Potential risk factors for the development of MTX-related toxicity were only identified in patient #1. She showed a pre-existing mildly impaired renal function (creatinine of 90 µmol/L and Chronic Kidney Disease Epidemiology Collaboration (CKD EPI) estimated Glomerular Filtration Rate (eGFR) of 65 mL/min/1.73 m^2^) and, therefore, received a reduced MTX dose (Table 1). She was also the only patient with a relevant third space. Pleural effusion was drained once prior to MTX administration and monitored thereafter without further fluid retention.

Chemotherapy was interrupted when HDMTX-associated toxicities became evident. In patient #3, treatment with pegylated asparaginase and 6-mercaptopurine was resumed on day 14 after start of MTX infusion when MTX had been cleared and renal function was restored. He was the sole patient to undergo MTX re-exposure and received two more cycles of dose-reduced HDMTX (33.3%) without any complications.

### Glucarpidase treatment

Glucarpidase was given in median 58 h (range 42–70 h) after MTX initiation (Table 1) and 34 h (19–38 h) after lab results triggering glucarpidase treatment. Drug administration was delayed due to logistics as in Germany glucarpidase is only available on individual patient request, being delivered as emergency supply from Belgium and the UK, respectively.

**Fig. 1:**
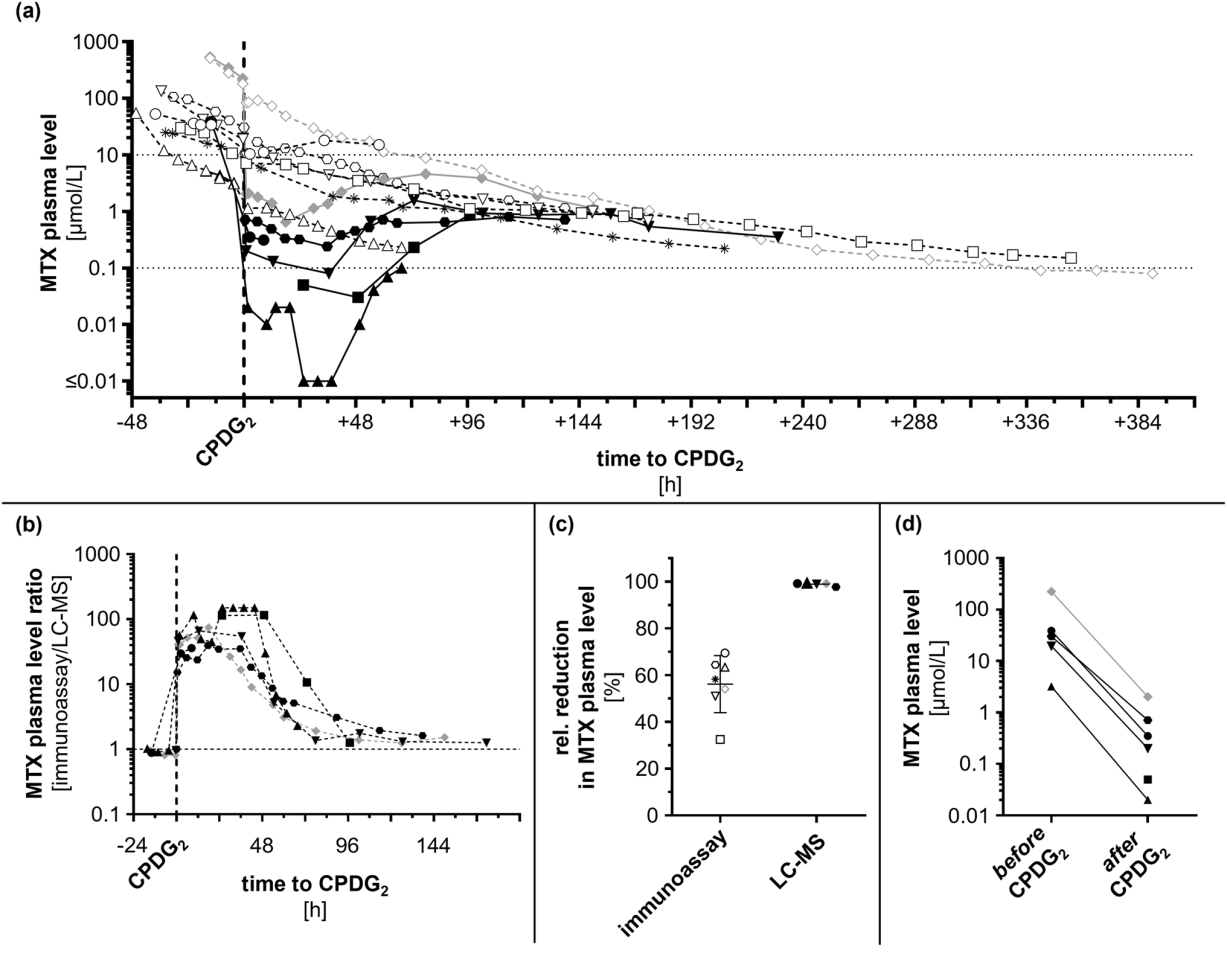
MTX plasma level. **a** MTX plasma level of individual patients determined by immunoassay (open shapes and dashed line) and by LC–MS (filled shapes and solid line) during glucarpidase treatment. **b** MTX plasma level ratio (immunoassay/LC–MS). **c** Relative reduction in MTX plasma levels comparing levels before and after glucarpidase as determined by immunoassay (left) and LC–MS (right). **d** MTX plasma levels as determined by LC–MS before (left) and after (right) glucarpidase. Patient #1 circle, #2 square, #3 upward triangle, #4 star, #5 downward triangle, #6 rhombus, #7 hexagon. Patient #6 who received dialysis is highlighted in gray. Abbreviations: *CPDG*_*2*_ glucarpidase, *LC–MS* liquid chromatography–mass spectrometry

RSCIR assessment was not possible [16], as in most patients MTX levels were neither determined immediately before (median 4.3 h, range 0–14.0 h) nor right after glucarpidase injection by LC–MS (median 1.3 h, 0.5–25.5 h) (Table 1 and Fig. 1A).

MTX levels simultaneously determined by immunoassay (in-house) and LC–MS (external) showed high accordance before glucarpidase treatment (pairwise measurements *n* = 9, ratio immunoassay/LC–MS mean = 0.92, range 0.81–1.02). As expected, immunoassay based MTX measurements provided falsely high results within 3 days after glucarpidase injection (pairwise measurements *n* = 42, ratio immunoassay/LC–MS min = 2.3) [20]. Later, both assays converged again (pairwise measurements *n* = 11, ratio immunoassay/LC–MS mean = 1.67, range 1.25–3.08) (Fig. 1B). A gradual rebound in MTX plasma levels as determined by LC–MS was detectable 24–73 h after glucarpidase.

By a single injection of half-dose glucarpidase, MTX plasma levels were reduced by ≥ 97.7% (Fig. 1C, right). MTX plasma levels < 10, < 1, and < 0.1 µmol/L at the first time point after glucarpidase injection were observed in 6, 5, and 2 patients with available LC–MS based measurements (*n* = 6), respectively (Fig. 1D).

### HDMTX-associated toxicities

**Fig. 2:**
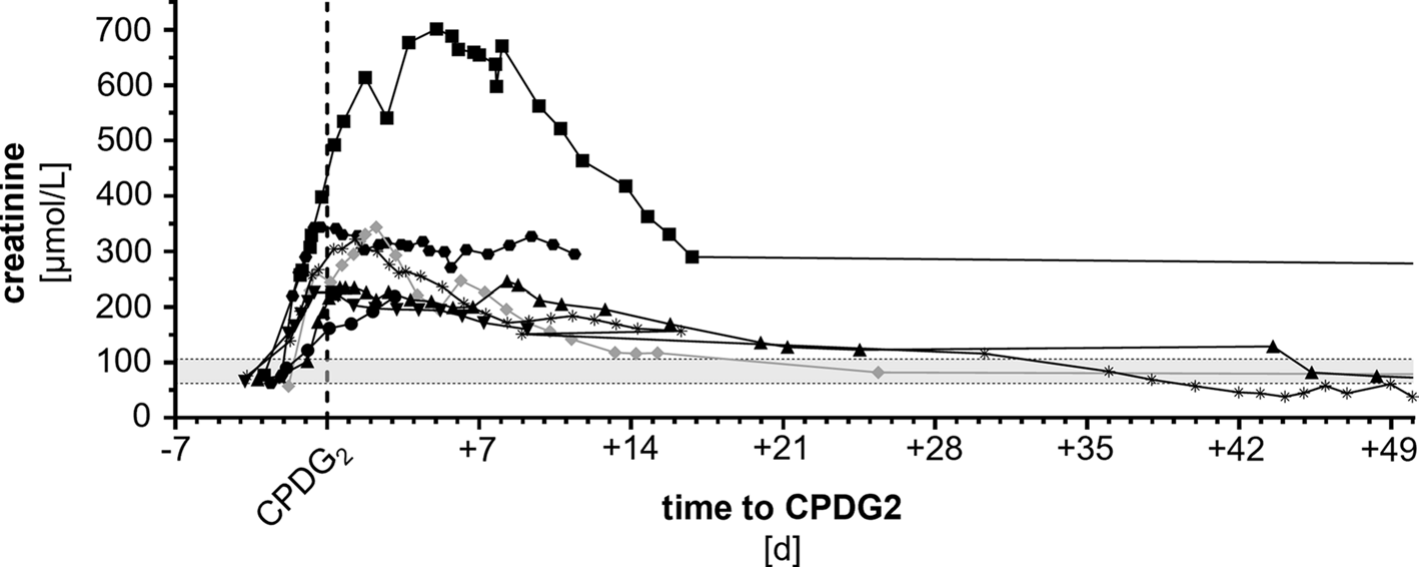
Creatinine levels. Renal function over time as depicted by creatinine levels in patients treated with half-dose glucarpidase because of high-dose MTX-associated acute kidney injury (gray shaded area represents reference range). Patient #1 circle, #2 square, #3 upward triangle, #4 star, #5 downward triangle, #6 rhombus, #7 hexagon. Patient #6 who received dialysis is highlighted in gray. *CPDG*_*2*_ glucarpidase

In patient #1–7, lab results triggering glucarpidase treatment first became evident at 24, 17, 36, 24, 24, 23, and 17 h after start of HDMTX infusion, respectively. Thus, all patients except #3 experienced significant increase in creatinine and toxic MTX levels within 24 h. Only patient #3 showed a slightly delayed AKI and toxic MTX levels occurring after 36 h (24 h: creatinine 102 µmol/L and MTX 55 µmol/L, 36 h: creatinine 173 µmol/L and MTX 12 µmol/L) (Table 1). In all patients except #1, urine excretion remained preserved during the observation period. According to the patient’s directive, no organ replacement was performed, and she died from progressive multiple organ failure 6 days after HDMTX initiation (3 days after glucarpidase administration); an autopsy was declined by the relatives. Patient #6 additionally received intermittent veno-venous hemodialysis (IVVHD) because of very high MTX levels and a delay in glucarpidase delivery. IVVHD was initiated 35.5 h after start of HDMTX infusion (6.5 h before glucarpidase) and paused 0.5 h before glucarpidase injection. IVVHD was resumed 80 h after glucarpidase and ultimately finished 3 days later when LC–MS based MTX levels became available. In patients without IVVHD (#1–5 and #7) creatinine levels peaked in median 83 h (range 48–144 h) after start of MTX and 19 h (− 6–97 h) after glucarpidase injection. Two patients (#1 and #7) died and one was discharged (#5) without creatinine level normalization. Patient #2 still showed a mildly to moderately impaired renal function after 2.5 years. Creatinine levels returned to baseline in patient #3, #4, and #6 at 46, 43, and 25 days after glucarpidase, respectively (Fig. 2).

Significant *hepatotoxicity* (≥ CTCAE grade 3) related to HDMTX occurred only in patient #6. Transaminases peaked between day 1 and 2 after MTX initiation and returned to ≤ grade 1 within 9 days.

*Thrombocytopenia* grade 4 on day 10 after start of MTX infusion was observed in patient #4, and platelet count (PLT) recovered to normal on day 19. Patient #7 also developed grade 4 thrombocytopenia on day 7, and PLT remained < 50 Gpt/L until his death 17 days after HDMTX initiation. Thrombocytopenia grade 3 occurred in patient #5 on day 9; PLT had increased to 58 Gpt/L on day 10 and the patient was lost to follow up. No patient developed major bleeding during the observation period.

Patients #3 and #7 temporarily showed *leukocytopenia* grade 3 on days 4–6 and day 4 after HDMTX initiation, respectively. Absolute neutrophil count of < 1 Gpt/L was only evident in patient #3 on day 6. Patient #1 and #7 suffered from infectious complications (#1 with pneumonia and #7 with gastrointestinal mucositis). All other patients remained free from infections and mucositis.

Three patients presented with intermittent impaired consciousness and dizziness. However, these symptoms were most likely attributable to the underlying CNS disease in two patients (#1 and #5) and did not represent MTX-associated *neurotoxicity*, as they were already present before HDMTX and did not substantially deteriorate during treatment. Only patient #7 presented with a newly emerging delirium. Computed tomography revealed progression of primary central nervous system lymphoma (PCNSL), identified as cause of death on day 17 of HDMTX; however, simultaneous MTX-associated neurotoxicity cannot be ruled out (Table 1).

## Discussion

Recently, an epidemiological study suggested that administration of glucarpidase may reduce mortality rates [21]. However, no randomized controlled study has formally proven that MTX level reduction by glucarpidase is associated with a favorable clinical outcome like decrease in MTX-induced toxicities, recovery of renal function or survival. The dose of 50 U/kg as recommended in the prescribing information has been shown to efficiently reduce MTX plasma levels which is used as surrogate parameter for clinical outcome [9, 14, 22, 23]. Few case series advocate that MTX plasma levels can be efficiently reduced even with glucarpidase doses lower than 50 U/kg [9–14]. Besides, dose reductions are a valuable mean to reduce the financial burden of this very costly treatment [13].

The influence on MTX-related neurotoxicity is a major concern as intravenous glucarpidase treatment does not affect MTX levels within the cerebrospinal fluid [24–27]. Therefore, half-dose glucarpidase is unlikely to improve symptoms of acute MTX-associated neurotoxicity. In our cohort, only one patient developed de-novo neurotoxicity, however, in the context of progressive PCNSL. Other toxicities were clinically manageable and limited in time. Only one patient with severe pre-existing comorbidities and a “do not intubate/do not resuscitate” order died early after initiation of MTX-based therapy because of multiple organ failure. Another patient died due to progressive disease. All other patients were discharged on average 18 days (± 2 days) post MTX without persistent clinically significant toxicities. Apart from one patient with sustained mildly to moderately impaired renal function, all other patients with longer observation periods recovered completely.

So far, Food and Drug Administration approval of glucarpidase solely relies on safety data and successful reduction of plasma MTX concentrations (RSCIR). In patients with pre-glucarpidase MTX plasma concentrations of > 1 to < 50 µmol/L, the RSCIR rate was 77% (*n* = 22), whereas patients with > 50 µmol/L did not achieve a RSCIR. The reason for RSCIR failure was mainly a transient rebound of MTX into the blood leading to plasma levels of > 1 µmol/L [16]. All patients in our cohort eventually fulfilled criteria for the use of glucarpidase as defined by consensus guidelines; however, two out of seven were not within the suggested time frame of ≤ 60 h after HDMTX initiation [8]. The efficacy assessment according to RSCIR criteria was not possible, but MTX concentrations of < 1 µmol/L were initially (median 1.3 h, 0.5–25.5 h after glucarpidase) observed in 83% of evaluable patients (5 out of 6) and four patients remained permanently < 1 µmol/L. However, all patients including the one who initially did not achieve MTX concentrations of < 1 µmol/L remained permanently at < 10 µmol/L. It has been suggested that only at MTX levels of > 10 µmol/L intensified FA rescue may be less effective [23, 28]. Clinical management of toxic MTX levels below this threshold might therefore be possible exclusively with prolonged intensified FA rescue. Some clinical reports even describe the feasibility of intensified FA rescue without excess in toxicity and mortality at much higher MTX levels [29, 30]. Therefore, half-dose glucarpidase seems to be sufficient to reliably achieve MTX concentrations manageable with intensified FA rescue.

Other publications have also dealt with the use of lower doses of glucarpidase. Eleven adult patients were reported who received glucarpidase at 10–31 U/kg because of a supply shortage. However, detailed efficacy data were not provided [7]. Other reports, mainly in pediatric patients, suggested that doses < 50 U/kg (minimum 7.5 U/kg) might be equally effective in lowering MTX levels [9–14]. Apart from the application of a fixed dose per bodyweight, it has been discussed to adjust glucarpidase to MTX concentration. The approved dose of 50 U/kg was proposed to be restricted to patients with moderately high MTX levels plus renal failure, whereas a reduction to 25 U/kg was suggested for only marginally high MTX concentrations. In patients with extremely high MTX levels (> 100 µmol/L), the glucarpidase dose might be increased to 100 U/kg [14]. In our cohort, half-dose glucarpidase lowered MTX in the only patient with > 100 µmol/L by 99.1%. Therefore, a desirable risk adapted glucarpidase application might be feasible even with lower dosages.

In conclusion, single half-dose glucarpidase efficiently reduced various MTX concentrations up to 182 µmol/L as determined by immunoassay. Levels obtained after glucarpidase administration were consistently < 10 µmol/L; therefore, manageable with continued intensified FA rescue. As randomized placebo controlled clinical studies to define the clinical effectiveness of glucarpidase are unlikely to be performed, at least dose finding studies including pharmacokinetic analysis and assessment of clinical outcome measures are urgently needed [21, 28]. Our study supports the use of glucarpidase below the recommended dose of the prescribing information which also translates into a significant economic benefit. In our cohort of seven patients, total cost savings were approximately 358,000 $. Recently, the price per vial was raised further, which means in a patient weighing 80 kg the use of half-dose glucarpidase reduces treatment expenditure by approximately 65,000 $; costs which are not routinely reimbursed by the health insurance in Germany.

## Supplementary Information

Below is the link to the electronic supplementary material.

Supplementary file1 (PDF 43 KB)

## Data Availability

All data generated or analyzed during this study are included in this published article.
