# Supplementary material for: Half-dose glucarpidase as efficient rescue for toxic methotrexate levels in patients with acute kidney injury
Source: Cancer Chemother Pharmacol. 2021 Oct 20;89(1):41–8. doi: 10.1007/s00280-021-04361-8 (PMC8739299; doi:10.1007/s00280-021-04361-8)
Supplement: Supplementary file 1 — Supplementary file1 (PDF 43 KB) [file 280_2021_4361_MOESM1_ESM.pdf]

Supplementary Information

Half-dose Glucarpidase as Efficient Rescue for Toxic Methotrexate Levels in Patients with Acute Kidney Injury

Cancer Chemotherapy and Pharmacology

Dr. Sandra Heuschkel[1], Dr. Theresa Kretschmann[2], Dr. Raphael Teipel[2], Dr. Simone von Bonin[2], Dr. Stephan Richter[2], Dr. Susanne Quick[4], Dr. Nael Alakel[2], Prof. Christoph Röllig[2], Dr. Ekaterina Balaian[2], Prof. Frank Kroschinsky[2], Dr. Holger Knoth[1], Prof. Martin Bornhäuser[2], Dr. Malte von Bonin[2,3,#]

[1] Klinik-Apotheke, Universitätsklinikum Carl Gustav Carus, Technische Universität Dresden (TUD), Fetscherstrasse 74, 01307 Dresden, Germany
[2] Medizinische Klinik und Poliklinik 1, Universitätsklinikum Carl Gustav Carus, Technische Universität Dresden (TUD), Fetscherstrasse 74, 01307 Dresden, Germany
[3] German Cancer Consortium (DKTK), partner site Dresden, Dresden, Germany, and German Cancer Research Center (DKFZ), Heidelberg, Germany
[4] Medizinische Klinik und Poliklinik 3, Universitätsklinikum Carl Gustav Carus, Technische Universität Dresden (TUD), Fetscherstrasse 74, 01307 Dresden, Germany
[#] Corresponding author: Dr. Malte von Bonin, ORCID ID 0000-0002-2995-3230

Supplemental Table 1: Folinic Acid Rescue in HDMTX (≥ 0.1 g/m^2 to ≤ 4 g/m^2 and ≤ 4 hours infusion time), warning signs and indicators for glucarpidase treatment

<table>
<tr><th>Time from start of MTX infusion [h]</th><th>Blood sampling (MTX level, creatinine)</th><th>Folinic acid adminis-tration</th><th>MTX level [µmol/L]</th><th>Folinic acid dose #</th><th>Warning signs for potentially protracted MTX clearance</th><th>Strong indicators for treatment with glucarpidase (modified from [8])</th></tr>
<tr><td>24</td><td>X</td><td>X</td><td></td><td>15 mg/m^2</td><td rowspan="3">MTX > 10 µmol/L or increase in creatinine >25% from baseline</td><td>MTX > 30 µmol/L ± creatinine > 25% from baseline</td></tr>
<tr><td>30</td><td rowspan="2"></td><td>X</td><td rowspan="3"><10
>10</td><td rowspan="3">15 mg/m^2
100 mg/m^2</td><td rowspan="3">MTX > 10 µmol/L ± creatinine > 50% from baseline</td></tr>
<tr><td>36</td><td>X</td></tr>
<tr><td>42</td><td>X</td><td>X</td><td>MTX > 10 µmol/L or increase in creatinine >50% from baseline</td></tr>
<tr><td>48</td><td>X</td><td>X</td><td rowspan="5"><0.1
≥0.1 to <1.0
≥1.0 to ≤5.0
>5.0</td><td rowspan="5">Stop rescue
15 mg/m^2
100 mg/m^2
MTX level x bodyweight [kg]</td><td rowspan="4">MTX > 1 µmol/L or increase in creatinine >50% from baseline</td><td rowspan="4">MTX > 5 µmol/L ± creatinine > 50% from baseline</td></tr>
<tr><td>54</td><td rowspan="3"></td><td>X</td></tr>
<tr><td>60</td><td>X</td></tr>
<tr><td>66</td><td>X</td></tr>
<tr><td>72</td><td>X</td><td>X</td><td rowspan="5">MTX > 0.1 µmol/L or increase in creatinine >50% from baseline</td><td rowspan="5"></td></tr>
<tr><td>78</td><td rowspan="3"></td><td>X</td><td rowspan="4"><0.1
≥0.1 to <0.4
≥0.4 to ≤5.0
>5.0</td><td rowspan="4">Stop rescue
15 mg/m^2
100 mg/m^2
MTX level x bodyweight [kg]</td></tr>
<tr><td>84</td><td>X</td></tr>
<tr><td>90</td><td>X</td></tr>
<tr><td>96</td><td>X</td><td>X</td></tr>
<tr><td>102</td><td rowspan="2"></td><td>X</td><td rowspan="2"></td><td rowspan="2"></td><td rowspan="2"></td><td rowspan="2"></td></tr>
<tr><td>108</td><td>X</td></tr>
<tr><td colspan="7">Continue blood sampling (MTX level and serum creatinine once a day) and folinic acid rescue every 6 hours as per time point t = 96 h until MTX <0.1 µmol/L</td></tr>
</table>

In the event of glucarpidase treatment, the dose of folinic acid should be reduced to 100 mg/m² after glucarpidase injection as LC/MS results are not available for dose calculation at short notice.

Supplemental Table 2: Folinic Acid Rescue in HDMTX (≥ 0.5 g/m^2 to ≤ 1.5 g/m^2 and 24 hours infusion time)[a], warning signs and indicators for glucarpidase treatment

Time from start of MTX infusion [h]	Blood sampling: MTX level	Blood sampling: creatinine	MTX level [µmol/L]	Folinic acid dose [#]	Warning signs for potentially protracted MTX clearance	Strong indicators for treatment with glucarpidase (modified from [8])
24	X	X	<150	-	MTX ≥150 µmol/L or increase in creatinine >25% from baseline	
			≥150	-		
36	X		<3	0 mg/m^2	MTX ≥3 µmol/L	MTX > 30 µmol/L ± creatinine > 50% from baseline
			≥3	30 mg/m^2		
42	X		<1	30 mg/m^2	MTX ≥2 µmol/L	MTX > 10 µmol/L ± creatinine > 50% from baseline
			≥1 to <2	30 mg/m^2		
			≥2 to <3	45 mg/m^2		
			≥3 to <4	60 mg/m^2		
			≥4 to <5	75 mg/m^2		
			≥5	MTX level x bodyweight [kg]		
48	X	X	<0.4	15 mg/m^2	MTX ≥1 µmol/L or increase in creatinine >25% from baseline	MTX > 5 µmol/L ± creatinine > 50% from baseline
			≥0.4 to <1	15 mg/m^2		
			≥1 to <2	30 mg/m^2		
			≥2 to <3	45 mg/m^2		
			≥3 to <4	60 mg/m^2		
			≥4 to <5	75 mg/m^2		
			≥5	MTX level x bodyweight [kg]		
54	X		<0.25	15 mg/m^2, then stop rescue	MTX ≥0.25 µmol/L	
			≥0.25 to <1	15 mg/m^2		
			≥1 to <2	30 mg/m^2		
			≥2 to <3	45 mg/m^2		
			≥3 to <4	60 mg/m^2		
			≥4 to <5	75 mg/m^2		
			≥5	MTX level x bodyweight [kg]		
Continue blood sampling (MTX level every 6 hours, serum creatinine once a day) and folinic acid rescue every 6 hours as per time point t=54 h until MTX <0.25 µmol/L						

[#] In the event of glucarpidase treatment, the dose of folinic acid should be reduced to 100 mg/m^2 after glucarpidase injection as LC/MS results are not available for dose calculation at short notice.

[a] GMALL 08/2013 trial (EudraCT 2013-003466-13)
